# P-241. Evaluating Acceptance of Anal Cancer Screening in HIV-positive MSM Veterans: A Real-World Study

**DOI:** 10.1093/ofid/ofaf695.463

**Published:** 2026-01-11

**Authors:** Minh Q Ho, Matthew Cole, Linda Chia, William Gage, Karen Slazinski, Mohammed Ahmed

**Affiliations:** Orlando VA Healthcare System, Orlando, FL; VA Capital Health Care Network VISN 5, Veterans Health Administration, Huntsville, Alabama; VA, Bellevue, Washington; Washington DC VA medical Center, Washington, District of Columbia; Orlando VA Healthcare System, Orlando, FL; Orlando VA Health Care system, Orlando, Florida

## Abstract

**Background:**

Anal cancer risk is dramatically elevated in people with HIV, particularly in men who have sex with men (MSM), bisexual men (BI), and transgender women (TGW). The ANCHOR trial showed that screening and treating high-grade squamous intraepithelial lesion (HSIL) can reduce anal cancer incidence by nearly 60%. In October 2024 IDSA Guidelines recommended annual anal cancer screening based on age in HIV positive patients. A report listing veterans over the age of 35 who identified as MSM, BI, TGW was added to the HIV Dashboard. The Veterans were offered ID pharmacist phone visits to discuss new changes. Here we report on patient acceptability of anal cancer screening at the Orlando VA Healthcare System.

**Figure:**
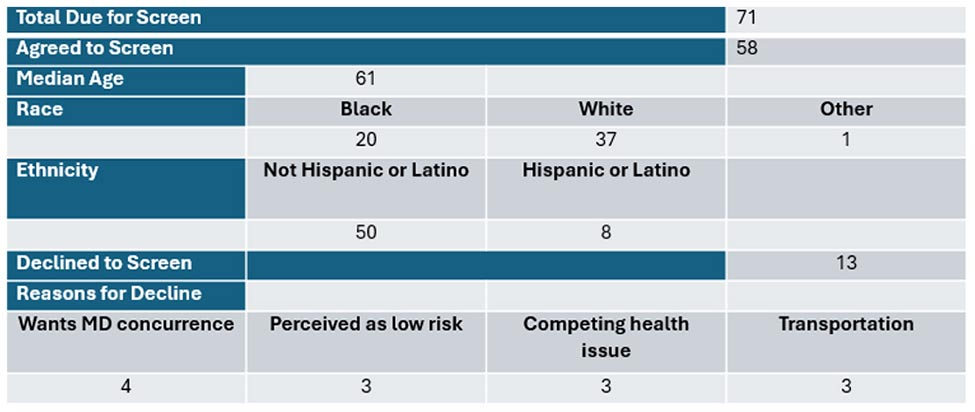
Characteristic of Eligible Veterans for Anal Cancer Screen

This table breakdowns the age, race and ethnicity of those Veterans due for anal cancer screening as well as the reasons for their decline

**Figure:**
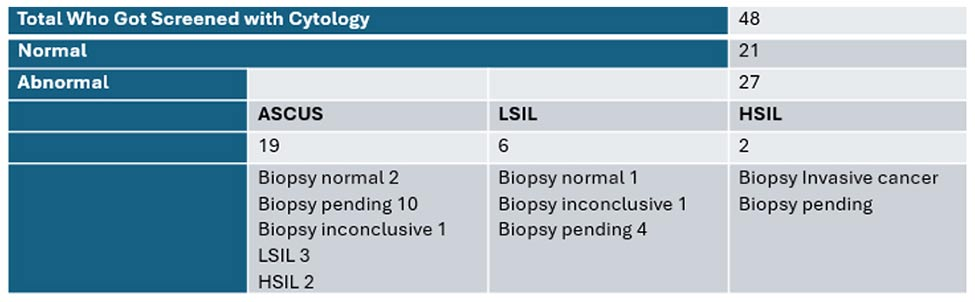
Description of Available Cytology

This table describes the cytology results of those who got screened as well as biopsy confirmation when available

**Methods:**

From January 01, 2025, to April 30, 2025, HIV-positive MSM, BI, or TGW Veterans over age 35 were offered an ID pharmacist phone appointment to review new HIV guidelines. For this quality improvement project, the ID pharmacist explained and offered proctology consults for anal cancer screening along with STI-3-site self-collection and vaccine orders when indicated. Data collected included patient name, age, acceptance of ID pharmacist phone appointment, number and percent of patients up to date with anal cancer screening, acceptance of anal cancer screening, completion of anal cancer screening, and results of anal cancer screening.

**Figure:**
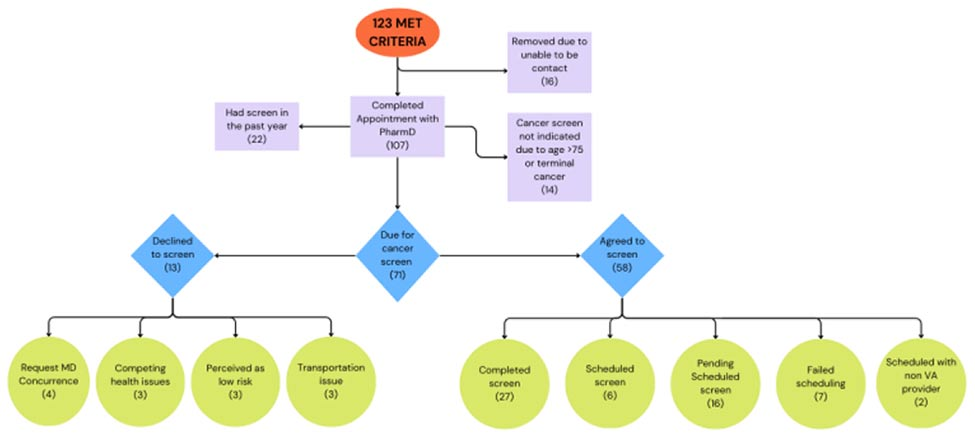
Flow diagram

This flow diagram describes who were eligible for anal cancer screening and subsequently got screened

**Results:**

A total of 123 veterans were identified, with 16 unreachable, resulting in 107 participants (87%) completing the phone visit. Of those, 71 (66%) were due for annual anal cancer screening, and 58 (81%) consented. 27 completed the screening, 6 scheduled future appointments, 16 accepted consults awaiting scheduling, 2 scheduled with non VA, and 7 failed the scheduling effort. Among the 48 individuals with cytology, 21 (44%) were normal, and 27 (56%) were abnormal. Of the abnormal, 19 were atypical squamous cells of undetermined significance (ASCUS), 6 were low-grade squamous intraepithelial lesion (LSIL), and 2 (HSIL).

**Conclusion:**

Veterans with HIV identifying as MSM, BI, or TGW showed high acceptance for phone consultations with pharmacists and a strong likelihood of agreeing to anal pap appointments. Other healthcare systems can use similar pharmacist-led strategies to improve adherence to anal cancer screening guidelines for sensitive topics.

**Disclosures:**

All Authors: No reported disclosures

